# Transient B Cell Depletion or Improved Transgene Expression by Codon Optimization Promote Tolerance to Factor VIII in Gene Therapy

**DOI:** 10.1371/journal.pone.0037671

**Published:** 2012-05-24

**Authors:** Brandon K. Sack, Sherin Merchant, David M. Markusic, Amit C. Nathwani, Andrew M. Davidoff, Barry J. Byrne, Roland W. Herzog

**Affiliations:** 1 Division of Cellular and Molecular Therapy, Department of Pediatrics, University of Florida, Gainesville, Florida, United States of America; 2 Powell Gene Therapy Center, University of Florida, Gainesville, Florida, United States of America; 3 Department of Haematology, UCL Cancer Institute, London, United Kingdom; 4 Department of Surgery, St. Jude Children's Research Hospital, Memphis, Tennessee, United States of America; Emory University School of Medicine, United States of America

## Abstract

The major complication in the treatment of hemophilia A is the development of neutralizing antibodies (inhibitors) against factor VIII (FVIII). The current method for eradicating inhibitors, termed immune tolerance induction (ITI), is costly and protracted. Clinical protocols that prevent rather than treat inhibitors are not yet established. Liver-directed gene therapy hopes to achieve long-term correction of the disease while also inducing immune tolerance. We sought to investigate the use of adeno-associated viral (serotype 8) gene transfer to induce tolerance to human B domain deleted FVIII in hemophilia A mice. We administered an AAV8 vector with either human B domain deleted FVIII or a codon-optimized transgene, both under a liver-specific promoter to two strains of hemophilia A mice. Protein therapy or gene therapy was given either alone or in conjunction with anti-CD20 antibody-mediated B cell depletion. Gene therapy with a low-expressing vector resulted in sustained near-therapeutic expression. However, supplementary protein therapy revealed that gene transfer had sensitized mice to hFVIII in a high-responder strain but not in mice of a low-responding strain. This heightened response was ameliorated when gene therapy was delivered with anti-murine CD20 treatment. Transient B cell depletion prevented inhibitor formation in protein therapy, but failed to achieve a sustained hypo-responsiveness. Importantly, use of a codon-optimized hFVIII transgene resulted in sustained therapeutic expression and tolerance without a need for B cell depletion. Therefore, anti-CD20 may be beneficial in preventing vector-induced immune priming to FVIII, but higher levels of liver-restricted expression are preferred for tolerance.

## Introduction

Approximately 25% of patients with the X-linked bleeding disorder hemophilia A (factor VIII, FVIII, deficiency) form inhibitory antibodies (inhibitors) to FVIII protein during replacement therapy. High-titer inhibitors (>5 BU) render treatment with FVIII impossible and necessitate the use of “bypassing” agents such as activated factor VII [1]. Inhibitors can be eradicated through a costly and protracted regimen known as “immune tolerance induction” (ITI). In ITI, patients are administered repeated high doses of FVIII for a period of up to 1–2 years. Although successful eradication occurs in >50% of patients, the cost can be upwards of $1,000,000 per patient [1], [2]. Thus, strategies to improve ITI or to induce tolerance to FVIII are highly desirable. One approach is through the use of the biologic drug rituximab-a monoclonal chimeric antibody directed against human CD20 originally developed to treat B cell lymphoma. Rituximab efficiently depletes CD20-expressing B cells via several mechanisms that include complement, antibody-mediated cellular cytotoxicity and direct induction of apoptosis [3]. CD20 is expressed from the early pre-B cell stage to mature B cells and short-lived plasma cells but not by long-lived plasma cells. Rituximab has been investigated for use in antibody-mediated autoimmune diseases such as acquired hemophilia, systemic lupus erythematosus, rheumatoid arthritis, multiple sclerosis, myasthenia gravis, and others [4]. Several case reports and one national survey have revealed that rituximab can improve ITI in hemophilia A patients, especially in cases where patients have previously failed traditional ITI [5]–[7]. Successful reversal of an inhibitor against factor IX (FIX) that had formed in a non-human primate after gene therapy was also reported using rituximab combined with cyclosporine A [8]. However, pre-clinical studies using αCD20 in hemophilic animals or in gene therapy for hemophilia are very limited. B cell depletion as a potential means of preventing (rather than reversing) inhibitor formation has also not been studied.

Interestingly, liver-directed gene therapy with adeno-associated virus (AAV) can provide both long-term phenotypic correction and immune tolerance to FIX in hemophilia B animal models [9]. Success in animal models has led to two clinical trials for hemophilia B using liver-directed AAV gene therapy [10]. Hemophilia A has been more difficult to treat with AAV gene therapy due to the increased immunogenicity of FVIII as well as limitations in the packaging capacity of AAV and in expression of FVIII. Typically, transient immune suppression, high vector doses, the use of canine FVIII (which has a higher specific activity in mice than hFVIII) and mice of C57BL/6 strain background (which is more promiscuous to hepatic AAV transduction), or a combination of these methods was needed to achieve long-term correction in hemophilia A mice [11]–[13]. Here, we investigate liver-directed AAV gene therapy in different strains of hemophilia A mice to induce tolerance to hFVIII either alone or in combination with transient B cell depletion.

## Results

### Combination of hepatic gene transfer and transient B cell depletion

**Figure 1:**
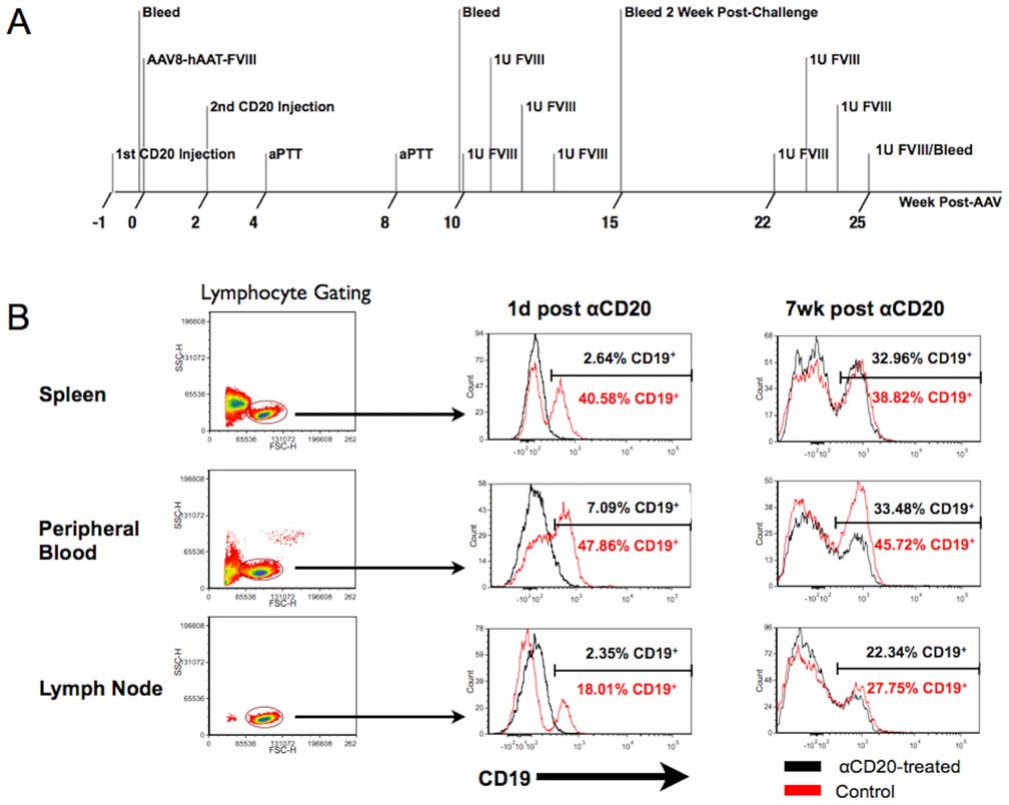
Depletion of B cells using anti-CD20. **A.** Treatment schedule. Hemophilia A (HA) mice in “αCD20” groups received a dose of 10 mg/kg IgG2a αCD20 i.v. on day 0 and day 21. One week following the first αCD20 injection, mice in “AAV8” groups received 10^11^ vg/mouse of AAV8-hFVIII. Blood samples were collected at indicated time points. Mice were challenged with intravenous hFVIII (1 IU/mouse, weekly for 4 weeks) at indicated time points. **B.** Representative examples of B cell depletion in different lymphoid organs of BALB/c-HA mice 1 day and 7 weeks after the second injection second αCD20 injection. Numbers in each histogram represent percent CD19^+^ lymphocytes (as shown by forward and side scatter gating in left panel) for both untreated control (in black) and αCD20-treated animals (red). Cells were stained with anti-CD19 antibody conjugated to V450 fluorochrome at 1 day post second injection, or to APC-Cy7 for the 7-weeks post-αCD20 time point.

This study sought to identify pathways toward immune tolerance to hFVIII in gene therapy and to determine the effect of transient B cell depletion on hFVIII-specific immune responses (using an anti-murine CD20 comparable to rituximab). Hemophilia A mice on either a mixed BL/6-129/sv (BL/6-129/sv-HA) or a BALB/c (BALB/c-HA) background were divided into two treatment groups (**Fig. 1A**). “AAV8-F8+αCD20” mice were given 10 mg/kg of αCD20 one week prior to receiving 10^11^vg/mouse of an AAV8 vector expressing B domain-deleted hFVIII under the liver-specific hAAT promoter (AAV8-F8). Two weeks following AAV8-hF8 injection (3 weeks following initial αCD20 injection), mice received another dose of αCD20. Mice in the group “AAV8-F8” received only the AAV8-hF8 vector (but not αCD20). Ten weeks following vector administration, all mice were challenged with weekly IV infusions of 1 IU hFVIII per mouse for 4 weeks (which reliably results in inhibitor formation in both strains). In case of BALB/c-HA mice, another 1-month challenge with weekly hFVIII injections was performed starting 22 weeks after gene transfer, and blood was collected within 15 min after the fourth hFVIII injection.

### Efficient B cell depletion with αCD20

B cell depletion in lymphoid organs was assessed 1 day and 7 weeks following the second αCD20 injection. CD19^+^ lymphocytes were largely absent from all sites investigated one day after the first or second αCD20 dose (**Fig. 1B** and data not shown for 1d post first injection). At 7 weeks after the second αCD20 dose (concurrent with initial hFVIII challenge), this B cell population had returned to normal levels in the spleen and lymph nodes and near normal levels in peripheral blood (**Fig. 1B**). Absolute T cell numbers, as measured by total CD3^+^ cells per 10,000 total cells in each organ, were not affected by treatment (data not shown). Thus, mice were deemed to be immune competent at time of hFVIII challenge.

### Lack of CD20^+^ B cells at the time of gene transfer renders BL/6-129/sv-HA mice hyporesponsive to hFVIII

**Figure 2:**
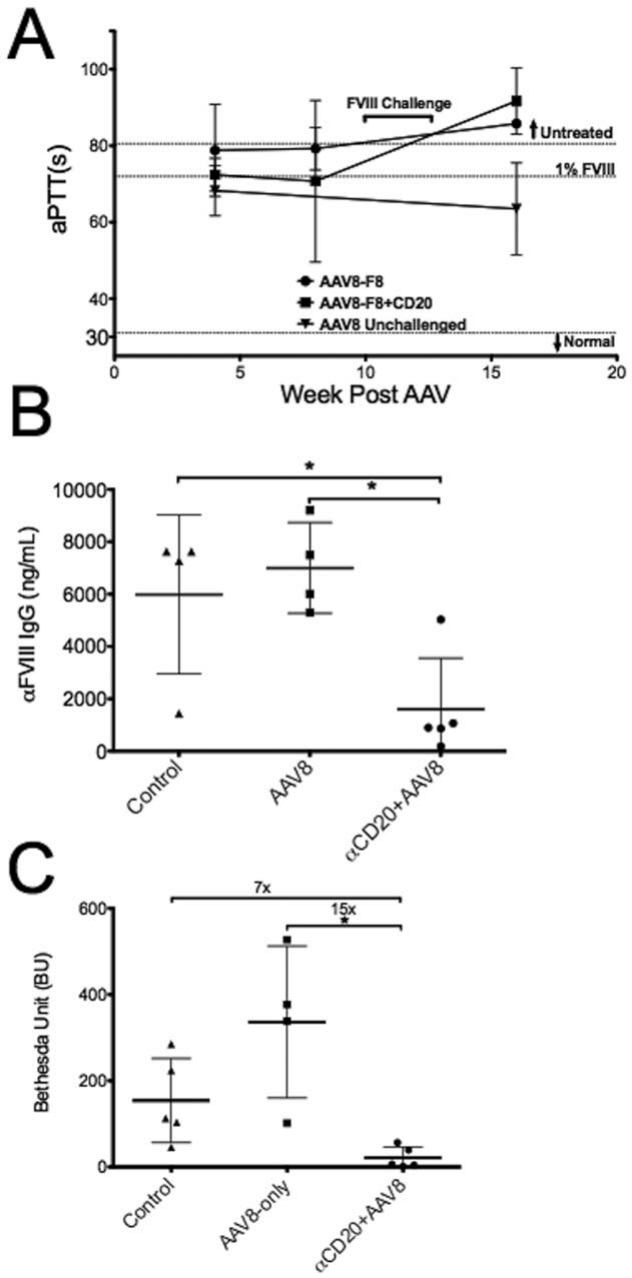
Depletion of B cells combined with gene therapy in BL/6-129/sv-HA mice. **A.** Activated partial thromboplastin time (aPTT) of mice receiving 10^11^vg/mouse of AAV8-hFVIII either alone (“AAV8-F8”) or in combination with αCD20 therapy (“AAV8-F8+CD20”). “FVIII challenge” indicates a period of weekly HFVIII injections (see Fig. 1A). “AAV8-unchallenged” group is mice that received vector but were not challenged with hFVIII protein. Range of aPTT for untreated mice and coagulation time for HA mouse plasma corrected to 1% HFVIII activity are also shown. Data are average values ±SD for n = 5 per experimental group. Two weeks after completion of the FVIII challenge, antibody formation against FVIII was measured: **B.** Total FVIII-specific IgG1 levels as determined by ELISA. **C**. Inhibitory antibody titers as measured by Bethesda assay (in BU). Values in panels B and C are shown for individual animals and as averages ±SD. Statistically significant differences between groups are indicated.

BL6-129/sv-HA showed modest correction of clotting times below that of untreated mice but this correction was minimal (≤1% of normal FVIII activity) regardless of αCD20 treatment (**Fig. 2A**). Without further manipulation, this level of correction was sustained for at least 4 months. However, those mice that were challenged with supplementary hFVIII therapy lost correction concurrent with the development of inhibitors (**Fig. 2C**). “Control” age-matched mice (no gene transfer) receiving identical hFVIII challenge had an average Bethesda titer of 154±44 BU, whereas mice receiving AAV8-hF8 had a higher average titer of 336±88 ng/mL, suggesting that AAV-hF8 treatment primed the mice against hFVIII. However, transient B cell depletion with αCD20 at the time of AAV8-hF8 gene transfer resulted in subsequent hyporesponsiveness to hFVIII with a significantly lower Bethesda titer (22±11 BU). These titers were 15 times lower than in mice receiving AAV8-hF8 without αCD20 and 7-fold lower than mice receiving only protein challenge. These results were also reflected in total circulating anti-hFVIII IgG1 as determined by ELISA (**Fig. 2B**). Mice in the “αCD20+AAV8-F8” group had a mean IgG1 titer of 1609±868 ng/mL, which was significantly lower than both control and AAV8-hF8 mice (which had titers of 5988±1520 ng/mL and 7001±867 ng/mL, respectively).

### AAV8-hFVIII gene transfer makes BALB/c-HA mice hyporesponsive to hFVIII regardless of B cell depletion

**Figure 3:**
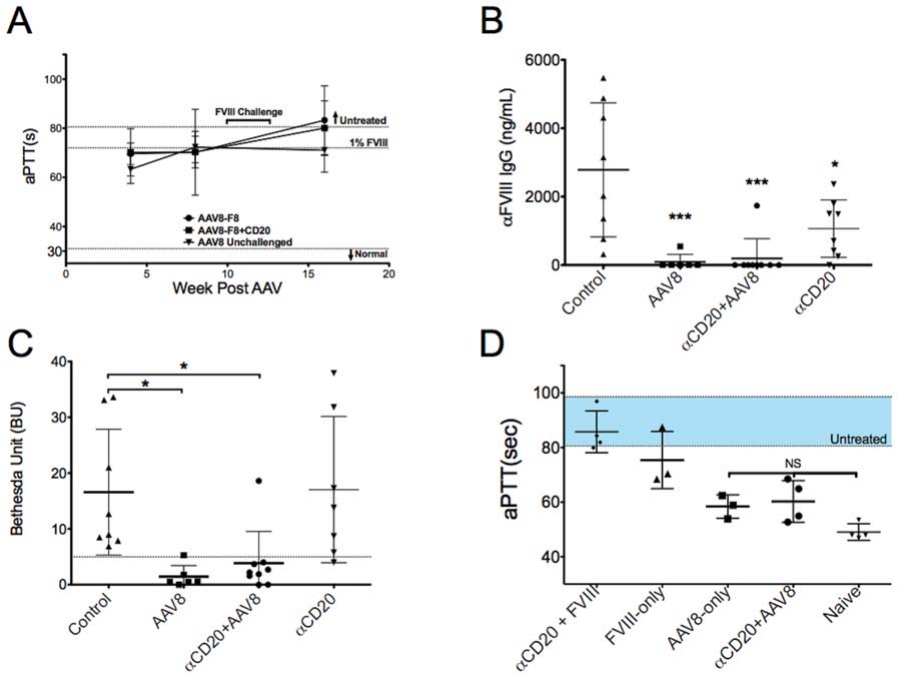
B Cell depletion combined with gene therapy in BALB/c-HA mice. **A.** Activated partial thromboplastin time (aPTT) of mice receiving 10^11^vg/mouse of AAV8-hFVIII either alone (“AAV8-F8”) or in combination with αCD20 therapy (“AAV8-F8+CD20”). “FVIII challenge” with weekly hFVIII protein is indicated (see also Fig. 1A). “AAV8-unchallenged” mice received vector but no hFVIII protein. “αCD20” mice received B cell depletion and hFVIII challenge in the absence of gene transfer. Range of aPTT for untreated mice and coagulation time for HA mouse plasma corrected to 1% HFVIIII activity are also shown. Data are average values ±SD for n = 7–9 mice per experimental group. Two weeks after completion of the FVIII challenge, antibody formation against HFVIIII was measured: **B.** Total FVIII-specific IgG1 levels. **C**. Inhibitor titers (in BU). Values in panels B and C are shown for individual animals and as averages ±SD. Statistically significant differences between groups are indicated. **D.** Starting in week 22, mice were given a second round of hFVIII protein challenge (see Fig. 1A). Five min after the last HFVIIII injection, mice were bled via tail vein, and collected plasma assayed by aPTT (n = 3–4 per experimental group). “Naïve” mice were also treated with hFVIII and bled 5 min later, but had no prior exposure to hFVIII. “FVIII-only” mice were mice that had been challenged previously with hFVIII (without αCD20 treatment or gene therapy) and thus had developed inhibitors. “αCD20+FVIII” mice had initially received αCD20 but no gene therapy and developed inhibitors in subsequent protein therapy (labeled as “αCD20” group in panels A–C). NS: not significant.

The above experiments were repeated in hemophilic mice carrying the same mutation on a BALB/c background. All BALB/c-HA mice receiving AAV8-hF8 had ∼1% FVIII activity, which was again sustained in the absence of further manipulation (**Fig. 3A**). Coagulation times of mice challenged with hFVIII increased to or near the uncorrected range. However, 8/9 mice in the “AAV8-F8+αCD20” group and 5/6 mice in the “AAV8-F8” group had at most low-titer inhibitors (<5 BU), and undetectable anti-hFVIII IgG1 (**Fig. 3B and 3C**). This was in contrast to control mice (no gene transfer or αCD20), which developed significantly higher anti-hFVIII IgG1 and significantly higher inhibitor titers (11-fold and 4-fold higher BU than “AAV-F8” and “AAV8-F8+αCD20” mice, respectively) (**Fig. 3B and 3C**). Another control group, “αCD20” mice, had received the same doses of αCD20 (but no gene transfer) followed by hFVIII challenge (following identical time lines). These mice developed similar Bethesda titers as the other control mice (**Fig. 3B**), albeit anti-FVIII IgG1 titers were somewhat lower (1067±297 ng/mL for “αCD20” vs. 2786±693 ng/mL for control; **Fig. 3C**).

We next wanted to determine if hyporesponsiveness would be maintained after another round of challenge, and if the supplementary exogenous hFVIII could correct the aPTT of vector-treated mice. The lack of humoral immune response was maintained in all but one mouse in the “AAV8-F8” group as measured by hFVIII-specific IgG (data not shown). In naïve BALB/c-HA mice, injection of 1 IU hFVIII resulted in correction of the aPTT to 49±2 sec at a 15-min time point (**Fig. 3D**). Mice in “AAV8-F8” and “AAV8-F8+αCD20” groups also showed correction with average clotting times of 58±3 sec and 60±4 sec, respectively. While this level of correction was not quite as good as for naïve mice, the difference did not reach statistical significance. No or marginal correction was achieved in control mice that had formed high-titer inhibitors (**Fig. 3D**). In summary, we conclude that treatment of BALB/c-HA mice with AAV8-hFVIII with or without B cell depletion confers a level of long-term tolerance to hFVIII that protects from high-titer inhibitors in subsequent protein therapy, thereby allowing for correction of coagulation.

### Induction of CD4^+^ T cell tolerance to hFVIII

**Figure 4:**
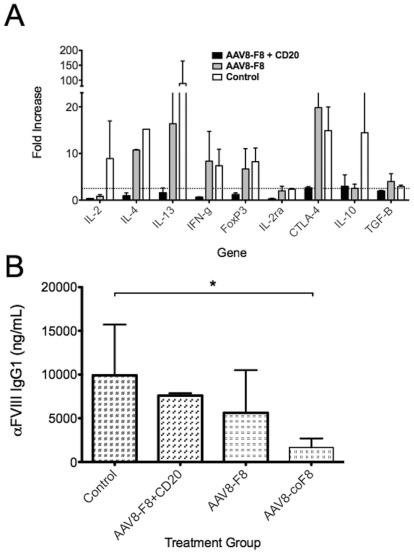
T cell responses in BALB/c-HA mice. **A.** Following the final tail vein bleed (Fig. 3D), mice were sacrificed and spleens collected. Splenocyte cultures for individual mice (n = 4 per group) were stimulated *in vitro* with 10 µg/mL of hFVIII for 48 h. Subsequently, CD3^+^CD4^+^ T cells were purified by flow cytometry and analyzed by quantitative RT-PCR for expression of several immune-regulatory genes. Shown are data for indicated groups (averages ±SD; “fold increase” is change in RNA transcripts of hFVIIII- vs. mock-stimulated). The dotted horizontal line indicates the minimally required increase of 2.5-fold for a statistically significant difference. **B.** Evidence for Treg induction in mice that had received gene transfer or a combination of αCD20 and gene transfer. Following one round of hFVIII challenge, CD4^+^CD25^+^ splenocytes were purified from each treatment group via magnetic sorting, and 10^6^ cells/mouse were adoptively transferred to naïve BALB/c-HA recipients via tail vein injection. Control cells were from unchallenged naïve mice of the same strain. Twenty-four hours later, all recipient mice (n = 3 per group) were challenged with 1 IU hFVIII in adjuvant. Anti-FVIII IgG titers were measured 1 month later by ELISA. Data are averages ±SD.

Inhibitor formation is dependent on T help and may be controlled by Treg. The BALB/c-HA strain was chosen for analyses of T cell responses to FVIII because this strain responded better to tolerance induction and, representing a pure strain background, could be used for adoptive transfer studies. Quantitative RT-PCR on *in vitro* hFVIII-stimulated CD4^+^ splenocytes was performed to test for induction of T cell tolerance. Responses in mice previously treated with AAV8-hFVIII+αCD20, AAV8-hFVIII only, or control mice (that had received protein challenge only) were measured after the last round of challenge with hFVIII protein had been completed. Bulk splenocytes were stimulated *in vitro* with hFVIII and subsequently sorted based on CD4 expression. RNA from CD4^+^ T cells was used in qRT-PCR to analyze the transcription of 9 different genes related to T cell immunity. CD4^+^ cells from control mice, which had developed high-titer inhibitors, showed increased transcription of a mix of Th1, Th2 and suppressive cytokine/marker genes including IL-2, IL-4, IL-13, IFN-γ, FoxP3, CTLA-4, and IL-10, and more modestly of TGF-β (**Fig. 4A**). Mice receiving AAV8-hFVIII alone showed a substantial reduction in their IL-2 and IL-10 responses and a partial reduction in IL-4 and IL-13 gene expression, while IFN-γ and also Treg markers remained similarly up-regulated as in controls. The “AAV8-F8+CD20” group showed a broad T cell unresponsiveness. Only IL-10 and CTLA-4 were marginally induced in response to FVIII.

The potential role of Treg in tolerance was further investigated by adoptive transfer of CD4^+^CD25^+^ cells harvested from “AAV8-F8+αCD20” or “AAV8-F8” mice after the first challenge only modestly suppressed anti-hFVIII formation in recipient mice (which did not reach statistical significance, Fig. 4B).

### hFVIII protein administration during recovery of B cells fails to achieve lasting tolerance

**Figure 5:**
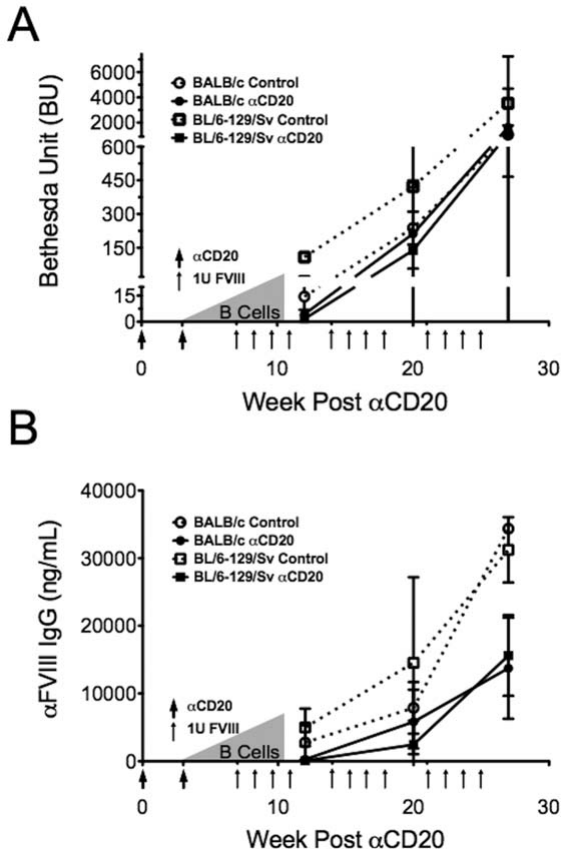
Anti-CD20 treatment to prevent antibody formation in hFVIII protein replacement therapy. BL/6-129/sv-HA and BALB/c-HA mice were treated with αCD20 antibody as outlined in Fig. 1A (indicated with large arrows) followed by 4 weeks of hFVIII challenge (indicated by small arrows) beginning at 4 weeks after the second αCD20 administration. Mice were treated with hFVIII twice more following the same schedule. Antibody formation against HFVIIII was measured by Bethesda assay (**A**) and anti-hFVIII IgG1 ELISA (**B**) two weeks after each 4-week challenge. Control mice did not receive αCD20. Gray triangles represent B cell recovery. Data are averages ±SD for n = 3–5/group.

Next we investigated whether B cell depletion could be combined with hFVIII protein administration to induce tolerance to hFVIII. Mice from each strain were given αCD20 as before at week 0 and 3. Starting at week 7 (i.e. 4 weeks past the last αCD20 dose), when B cells were still recovering, mice were given 4 weekly IV doses of 1 IU of hFVIII. Mice were bled 2 weeks later to test for antibody formation. Initially, mice treated with αCD20 were either non-responsive or hyporesponsive to challenge. Only 1 of 8 BL/6-129/sv-HA mice had detectable anti-FVIII antibodies (513 ng/mL; 3 BU), whereas 3/7 BALB/c-HA mice had detectable (albeit low-titer) inhibitors (**Fig. 5A,B**). As expected, control mice (no αCD20 treatment) developed high-titer inhibitors. The hFVIII treatment protocol was repeated at weeks 14–17 and 21–24. After the second challenge, only 2 of the BL/6-129/sv-HA mice remained unresponsive, whereas all other mice developed an antibody response similar to controls after the first challenge. As expected, control mice showed further increases in both IgG and Bethesda titers. By the third challenge, all mice treated with αCD20 had developed a humoral immune response to hFVIII similar in magnitude to the controls after two challenges (**Fig. 5A,B**). Therefore, transient B cell depletion merely delayed the antibody response to hFVIII protein.

### Higher hepatic expression improves efficacy and tolerance induction

**Figure 6:**
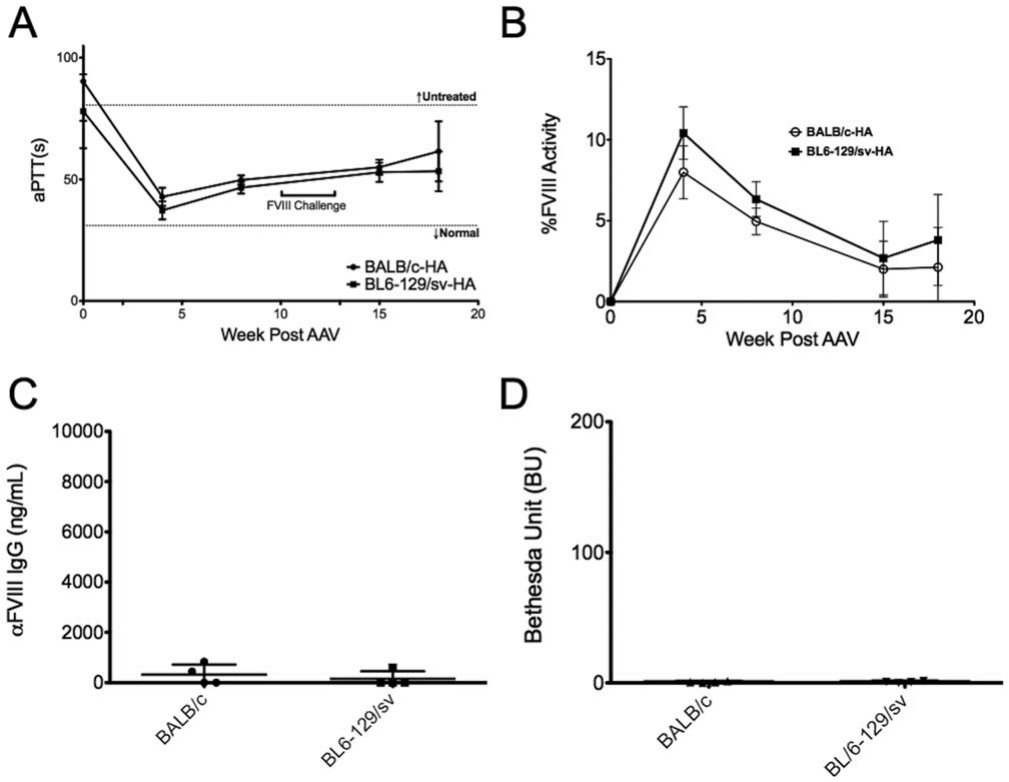
Codon-optimized hFVIII to induce tolerance and correction. BALB/c-HA and BL/6-129/sv-HA mice were injected with 10^11^vg/mouse of an AAV8 vector expressing codon-optimized hFVIII. **A.** Coagulation times (aPTT in sec) and **B.** HFVIIII activity were measured as a function of time after vector administration. Data are averages ±SD for n = 4/group. Mice were challenged (starting at week 10 after gene transfer) with hFVIII at the same dose and schedule as in previous experiments. Anti-hFVIII formation was measured **C.** as HFVIIII-specific IgG1 titers, and **D.** by Bethesda assay. Values in panels C and D are shown for individual animals and as averages ± SD and plotted on the same scale as in Fig. 2 to compare magnitude of responses.

We also tested an AAV8 vector containing codon-optimized hFVIII (AAV8-cohFVIII). Identical doses of this vector resulted in drastic improvements of clotting times in both strains, with average aPTTs declining from 78±8 sec to 37±2 sec in the BL/6-129/sv-HA mice and from 90±8 sec to 43±2 sec in BALB/c-HA mice after 4 weeks (**Fig. 6A**). Clotting times generally rose over the following 4 weeks but then stabilized. Means of 53±4 sec in BL/6-129/sv-HA and 61±6 sec for BALB/c-HA mice were measure at 18 weeks. Initially, hFVIII activities were 10±1% and 8±1% in BL/6-129/sv-HA and BALB/c-HA mice, respectively, with a decline to 4±1% and 2±1% (**Fig. 6B**). Following challenge with hFVIII protein, 3 of 4 mice in the BL/6-129/sv showed no antibody response to hFVIII with the other having a muted IgG1 titer of 603 ng/mL compared with an average of 5988±3040 ng/mL in control animals challenged in the same manner (**Fig. 6C**). This animal also had a low Bethesda titer of 1.4 BU (**Fig. 6D**), resulting in a longer aPTT (64.9 sec) compared to the other mice at 18 weeks post vector injection. In BALB/c-HA mice, no anti-hFVIII antibodies were detected in 2/4 mice, while the other 2 mice had very low but detectable antibody titers after challenge (830 and 443 ng IgG1 anti-hFVIII/mL, corresponding to 1.4 and 0.6 BU, **Fig. 6C,D**). Average anti-FVIII titers of the control group were 2785±693 ng/mL or 17±5 BU. In summary, a highly significant 9–10-fold reduction in antibody response to hFVIII protein was achieved with the cohFVIII construct including a complete lack of response to hFVIII protein in 5/8 mice.

Adoptive transfer of CD4^+^CD25^+^ Treg was also performed as above with tolerant AAV8-coFVIII-treated BALB/c-HA mice serving as donors. Recipient mice showed a significantly lower antibody response to FVIII compared to mice receiving CD4^+^CD25^+^ T cells from naïve mice (**Fig. 4B**). Therefore, the use of cohFVIII induced a more potent Treg response than wild-type cDNA.

## Discussion

Given the recent success with hepatic AAV8 gene transfer in patients with severe hemophilia B, a similar gene therapy for hemophilia A represents a logical progression [14]. However, therapeutic AAV-FVIII gene transfer in animal models has required substantially higher vector doses and the risk of inhibitor formation is generally greater for hemophilia A. We previously demonstrated tolerance induction to FIX by AAV gene transfer to the liver of hemophilic animals, which was particularly effective when using AAV8-a serotype that is highly effective in transducing murine hepatocytes [9], [15]. However, a minimal level of transgene expression was required.

### Gene transfer may tolerize or sensitize mice depending on genetic background

If gene transfer only accomplished expression at the lower end of the therapeutic range, e.g. <5% of normal, this would occasionally necessitate supplementary clotting factor administration. Hence, it is imperative that tolerance is maintained under these circumstances. Previously, sustained correction of murine hemophilia A with AAV vectors has been shown using either cFVIII or murine FVIII, but neither study tested the effect of FVIII challenge after gene therapy [16], [17]. Others have combined gene transfer with immune suppression to avoid inhibitor formation [11]–[13], [16]. Our study shows that lack of inhibitor formation against FVIII after hepatic AAV gene transfer does not necessarily constitute humoral immune tolerance as mice in both strains treated with a low-expressing vector maintained correction in absence of but not after challenge with hFVIII (**Figs. 2A** **and** **3A**). In fact, in the BL/6-129/sv-HA strain, mice were predisposed to heightened inhibitor formation in subsequent treatment with hFVIII protein, while gene transfer had the desired tolerogenic effect in BALB/c-HA mice. Clearly, high levels of hepatic expression promote tolerance regardless of the strain background (Fig. 6), which is consistent with our earlier findings on factor IX [9]. However, within the range of sub-optimal expression, two possibilities may explain the differing responses between the two strains. Somewhat higher expression in BALB/c-HA mice may have been partially effective in tolerance induction, while expression in the BL/6-129/sv-HA strain may have been just below this threshold and thus sensitized the mice. Alternatively, similarly low levels of expression may sensitize animals with higher immune responsiveness to F.VIII, while having a more tolerogenic effect in a strain with lower responsiveness.

Consistent with data by others, BALB/c-HA mice had lower inhibitor responses against hFVIII, and T helper responses differed between strains [18]. BL/6-129/sv-HA mice, after 4 weeks of hFVIII protein therapy, had 10-fold higher inhibitor titers compared to BALB/c-HA. However, IgG formation against hFVIII was only 2-fold higher (compare **Figs. 2** **and** **3**). In contrast to BL/6-129/sv-HA, BALB/c-HA mice only showed a good correlation between BU and IgG titers after a longer course of hFVIII administration (data not shown). Previously, we documented that CD4^+^ T cell responses against hFVIII in BL/6-129/sv-HA mice were strongly biased to Th2, characterized by IL-4 and IL-6 production while lacking expression of suppressive/regulatory cytokines [19]. BALB/c-HA mice, however, have a more mixed response, which includes expression of Th1, Th2, and suppressive molecules (**Fig. 4A**). This difference in the T helper response likely explains differences in inhibitor responses and in ability to induce tolerance in the 2 strains. Despite evidence for Th1 activation as part of the CD4^+^ T cell response in BALB/c-HA, we found that mice of both strains formed IgG2a against hFVIII only rarely and inconsistently (data not shown), suggesting that antibody formation is primarily driven by the Th2 component, thus consistently resulting in high-titer IgG1.

### Tolerance induction with codon-optimized hFVIII

It is encouraging that a more effective codon-optimized hFVIII expression cassette induced humoral tolerance, resulting in sustained correction of coagulation after challenge with hFVIII protein, independent of the strain background. This is significant in light of the fact that hFVIII has been notoriously difficult to express in mice at levels high enough for phenotypic correction. Often, alternative species FVIII have been used such as canine FVIII or porcine FVIII because of either higher expression or superior secretion [13], [17], [20], [21]. Here, we were able to achieve tolerance to hFVIII and long-term phenotypic correction with a single AAV vector expressing codon-optimized hFVIII in the absence of immune suppression. Codon optimization is a process whereby the cDNA to be expressed is analyzed and altered to improve expression based on a variety of factors including but not limited to: usage of species-specific codons, increased GC content, removal of cryptic slice sites, and stabilizing RNA structure [22]. This process has been used in the context of gene therapy to improve expression of several transgenes from different vectors [23]–[28]. Only one study used this approach for hFVIII expression. Investigators observed up to a 44-fold increase in FVIII expression following delivery of a codon-optimized hFVIII via lentivirus to neonatal hemophilia A mice [28]. However, the immunological consequences of increased expression in the context of a mature immune system were not investigated. Here, we demonstrate that a cohFVIII construct improves not only efficacy but also tolerance induction in adult hemophilia A mice of two different strains. In adult animals, Treg induction is a critical component of tolerance induction by hepatic gene transfer. Our adoptive transfer experiments showed that use of cohFVIII facilitated Treg induction, thereby conferring better suppression of antibody formation against hFVIII.

### Utility of transient B cell depletion for tolerance to hFVIII

Rituximab has been used to treat patients with inhibitors with varying degrees of success [5]–[7]. In acquired hemophilia, a rare form of the disease where inhibitors appear in a hemostatically normal individual, treatment with rituximab alone can eradicate inhibitors [29]. This response is long-lasting and has been observed to last up to 5.5 years [29]. In contrast, rituximab use in X-linked hemophilia A is usually concurrent with traditional ITI. In this setting, data is limited largely to case reports and well-controlled studies are lacking. However, a consecutive national cohort analyzed in the UK investigating the use of rituximab as an adjunct to traditional ITI suggests a modest benefit of B cell depletion for eradicating inhibitors in patients who have previously failed ITI [6]. Still, a number of these individuals (6/10) relapsed and required additional immune therapy [6]. Our data presented here also reveal limitations for the use of αCD20. While B cell depletion effectively prevented anti-hFVIII formation in protein therapy, such a protocol failed to induce lasting hyporesponsiveness, suggesting that protein administration fails to tolerize the newly emerging B cells. This conclusion is supported by a recent clinical study where immune responses to neoantigens following Rituximab treatment were muted during B cell depletion but returned to near normal once the effects of the drug waned [30]. Furthermore, our experiments using B cell depleting αCD20 in conjunction with gene therapy suggest its usefulness when there is concern of low-efficacy gene therapy priming the immune system to hFVIII. This indicates a role of B cells in the generation of a primary immune response to hFVIII. This was not due to long-term effects of αCD20 as transient B cell depletion did not reduce the ability of control animals to form antibodies once B cell frequencies had recovered. To further confirm this point, we administered an adenoviral vector expressing β-galactosidase 7 weeks after the second dose of αCD20. The mice formed neutralizing antibodies against the virus and antibodies against β-galactosidase at titers indistinguishable from control mice (data not shown).

Low levels of hepatic expression resulted only in a partial reduction of the T cell response to FVIII in BALB/c-HA mice, despite the protective effect against inhibitor formation. The reduction of the Th2 response, in conjunction with preserving Treg responses, was likely instrumental in achieving this effect. Nonetheless, gene transfer combined with αCD20 treatment induced a broad T cell unresponsiveness. Interestingly, up-regulation of Treg markers was also abrogated. This was somewhat reflected in adoptive transfer experiments, where CD4^+^CD25^+^ cells from AAV8-F8-only treated mice showed a trend for greater suppression compared to cells from “AAV8-F8+αCD20” mice, although this did not reach significance. In contrast, high expression from the codon-optimized construct increased the suppressive response in the presence of B cells, which raises the question of how the combination of these two approaches may shape the response. B cells play a dual role in the immune response as antigen presenting cells and as producers of antibodies. Zhang *et al.* demonstrated in mice that treatment with αCD20 similarly prevented a secondary immune response to protein challenge in hFVIII-primed hemophilia A mice [31]. Interestingly, these authors found that IgG1 instead of IgG2a αCD20 spared marginal zone B cells and caused accumulation of Treg after further exposure to hFVIII. Furthermore, a recent study by Xiang *et. al* investigating the effect of B cell depletion on T cell function and phenotype [32]. In their model, Treg were initially reduced following B cell depletion but actually increased when B cells have recovered, and there was also evidence for emergence of suppressive B cells. Thus, a more differential method for B cell targeting may further improve tolerance induction by this approach.

In conclusion, transient B cell depletion and even more so use of a codon-optimized FVIII sequence in hepatic gene transfer represent promising strategies to avoid inhibitor formation and promote tolerance in gene therapy for hemophilia A. Therefore, adding a different and superior method for tolerance induction such as hepatic gene transfer may improve the usefulness of rituximab for elimination of inhibitors in congenital hemophilia. Going forward, it will be critical to determine whether transient B cell depletion combined with use of a codon-optimized expression cassette synergize in inducing tolerance to hFVIII, and what the effect on T effector and Treg responses will be. In addition, anti-CD20 treatment may facilitate both tolerance to the transgene product and re-administration of vector by preventing neutralizing antibodies against the vector capsid.

## Methods

### Ethics statement

This research involved hemophilic mice. These studies (Approval ID # 01321, “Immunology of factor IX gene transfer to liver”, and Approval ID # 03971, “Pathways towards immune tolerance to coagulation factors”) were approved by the respective Institutional Animal Care and Use Committees (IACUC) at the University of Florida and the NIDCR (NIH). All procedures were done in accordance with the principles of the National Research Council's Guide for the Care and Use of Laboratory Animals. Studies were conducted in male mice housed at 22–24°C in a 12 hours light/dark cycle. Mice had free access to water and food unless indicated otherwise.

### Mouse strains

All animals used were 6–10-week old male hemophilia A mice with a deletion in Exon 16 of the mFVIII gene on either BALB/c or a mixed C57BL/6-129/sv background, kindly provided by Drs. Lillicrap and Kazazian [33].

### B cell depletion and flow cytometry

Mice were depleted of B cells using 10 mg/kg of anti-murine CD20 IgG2a antibody (clone 18B12, kindly provided by Biogen Idec) [34]. Mice were given two IV doses 21 days apart. To assess B cell depletion, lymphoid tissues were collected either 1 day or 7 weeks after the second dose. Peripheral blood was collected in heparinized microcapillary tubes via retro-orbital plexus, centrifuged to remove plasma, and remaining cells suspended in PBS for flow cytometry. Single cell suspensions of splenocytes and peripheral lymph node cells were stained with fluorescently labeled antibodies for the B cell marker CD19 as well as CD3 or CD4 to detect T cells. Lymphocyte gating was determined by forward-scatter and side-scatter following back gating for the area containing CD19^+^ cells. A BD LSRII flow cytometer (BD Biosciences, Frederick, MD, USA) and FCS Express 4 software (Denovo Software, Los Angeles, CA, USA) were used for analysis.

### Vectors and mouse procedures

Mice were given a tail vein injection of 10^11^vg/mouse of AAV8 containing either wild-type or codon-optimized B domain-deleted human hFVIII (BDD-hFVIII) under a liver-specific promoter. This construct was assembled by replacing the transthyretin promoter of the vector published by Lu *et al.* [12] with the ApoE/hAAT enhancer/promoter [9]. The codon-optimized cDNA was synthesized by GeneArt (Regensburg, Germany). Tail bleeds as described previously [35]. For hFVIII challenge, mice were administered 1 IU BBD-hFVIII (Xyntha, Pfizer, New York, NY) once a week for 4 weeks beginning at week 10 after vector administration. Collected blood was used to determine activated partial thromboplastin time (aPTT), while antibody responses were measured via ELISA for total anti-hFVIII IgG and by Bethesda assay as described [35]. For hFVIII % activity, a standard curve was developed using the same BDD-hFVIII used for challenge diluted in naïve hemophilia A mouse plasma.

### T cell assays and adoptive transfer

Splenocytes were isolated from mice in each group and cultured in RPMI 1640 media containing 5 µM β-mercaptoethanol, 100 mM insulin/transferrin/selenium, glutamine and pencillin/streptomycin either with or without 5 µg/mL of hFVIII for 48 h at 37°C and 5% CO_2_. Cells were then removed and sorted for CD3^+^CD4^+^ cells on an ARIA cell sorter (BD Biosciences, San Jose, CA, USA). RNA was immediately extracted from these cells using the Qiagen RNeasy isolation kit (Valencia, CA, USA) and used for quantitative RT-PCR with SA Biosciences arrays. Splenic CD4^+^CD25^+^ cells were purified with the magnetic isolation kit from Miltenyi Biotec (Bergisch Gladbach, Germany), which achieved ∼85% purity. These cells were then pooled and adoptively transferred to naïve BALB/c-HA mice at 10^6^ cells/mouse via tail vein injection. Recipient mice were challenged 24 hours later via subcutaneous injection of 1 IU hFVIII in Sigma Adjuvant System (St. Louis, MO, USA).
